# Impact of Remote Titration Combined With Telemonitoring on the Optimization of Guideline-Directed Medical Therapy for Patients With Heart Failure: Internal Pilot of a Randomized Controlled Trial

**DOI:** 10.2196/21962

**Published:** 2020-11-03

**Authors:** Veronica Artanian, Heather J Ross, Valeria E Rac, Mary O'Sullivan, Darshan H Brahmbhatt, Emily Seto

**Affiliations:** 1 Institute of Health Policy, Management and Evaluation Dalla Lana School of Public Health University of Toronto Toronto, ON Canada; 2 Ted Rogers Centre for Heart Research Peter Munk Cardiac Centre University Health Network Toronto, ON Canada; 3 Centre for Global eHealth Innovation Techna Institute University Health Network Toronto, ON Canada; 4 Department of Medicine University of Toronto Toronto, ON Canada; 5 Toronto Health Economics and Technology Assessment Collaborative University Health Network Toronto, ON Canada; 6 Toronto General Hospital Research Institute University Health Network Toronto, ON Canada; 7 Peter Munk Cardiac Centre Division of Cardiology University Health Network Toronto, ON Canada; 8 National Heart and Lung Institute Imperial College London London United Kingdom

**Keywords:** telemonitoring, remote, titration, monitoring, mHealth, heart failure

## Abstract

**Background:**

To improve health outcomes in patients with heart failure, guideline-directed medical therapy (GDMT) should be optimized to target doses. However, GDMT remains underutilized, with less than 25% of patients receiving target doses in clinical practice. Telemonitoring could provide reliable and real-time physiological data for clinical decision support to facilitate remote GDMT titration.

**Objective:**

This paper aims to present findings from an internal pilot study regarding the effectiveness of remote titration facilitated by telemonitoring.

**Methods:**

A 2-arm randomized controlled pilot trial comparing remote titration versus standard care in a heart function clinic was conducted. Patients were randomized to undergo remote medication titration facilitated by data from a smartphone-based telemonitoring system or standard titration performed during clinic visits.

**Results:**

A total of 42 patients with new-onset (10/42, 24%) and existing (32/42, 76%) heart failure and a mean age of 55.29 (SD 11.28) years were randomized between January and June 2019. Within 6 months of enrollment, 86% (18/21) of patients in the intervention group achieved optimal doses versus 48% (10/21) of patients in the control group. The median time to dose optimization was 11.0 weeks for the intervention group versus 18.8 weeks for the control group. The number of in-person visits in the intervention group was 54.5% lower than in the control group.

**Conclusions:**

The results of this pilot study suggest that remote titration facilitated by telemonitoring has the potential to increase the proportion of patients who achieve optimal GDMT doses, decrease time to dose optimization, and reduce the number of clinic visits. Remote titration may facilitate optimal and efficient titration of patients with heart failure while reducing the burden for patients to attend in-person clinic visits.

**Trial Registration:**

ClinicalTrials.gov NCT04205513; https://clinicaltrials.gov/ct2/show/NCT04205513

**International Registered Report Identifier (IRRID):**

RR2-10.2196/preprints.19705

## Introduction

### Background

Heart failure (HF) is a progressive condition with periods of stability interrupted by periods of worsening symptoms and instability, often leading to hospitalization [1]. The Canadian Cardiovascular Society distinguishes between HF with preserved ejection fraction (left ventricular ejection fraction [LVEF] ≥50%), HF with midrange ejection fraction (LVEF 41%-49%), and HF with reduced ejection fraction (HFrEF) (LVEF ≤40%) [2]. HFrEF is a distinct group in which large clinical trials have demonstrated the efficacy of neurohumoral inhibition [3]. 

The recommended therapeutic approach for patients with HFrEF consists of triple therapy with angiotensin-converting enzyme inhibitors (ACEIs), angiotensin receptor blockers (ARBs), or angiotensin receptor–neprilysin inhibitors (ARNIs); β-blockers (BBLs); and mineralocorticoid receptor antagonists (MRAs) [4-6]. These medications have been shown to improve symptoms, reduce hospitalization burden, and provide survival benefit in randomized controlled trials (RCTs) [6-10]. Titration of guideline-directed medical therapy (GDMT) to doses proven effective in clinical trials or maximally tolerated doses is recommended to reduce morbidity and mortality [2]. Expert recommendations suggest that clinicians should aim to achieve target doses within 3 to 6 months of initial HF diagnosis [11].

Despite proven benefits and strong guideline recommendations, large registries confirm that GDMTs are underutilized, and management of HF tends to fall short in respect to dose optimization [12-14]. In clinical practice, evidence from 12,440 patients with HF on the European Society of Cardiology Heart Failure Long-Term Registry showed that about 30% of patients were on target doses of ACEIs and only 18% were on target doses of BBLs [15]. Similarly, the Change the Management of Patients with Heart Failure (CHAMP-HF) registry, which analyzed data from 3518 patients in the United States, revealed that at baseline, only 16.7% of patients were on target doses of ACEIs, ARBs, or ARNIs, 27.5% were on target doses of BBLs, and 76.6% were on target doses of MRA therapy [13].

Evidence-based pharmacotherapies have the greatest potential to improve population-level outcomes, as they are less costly and more easily accessible than devices and surgical procedures available to patients with HF [12]. This reinforces the need to find ways to improve adherence to GDMT. Remote titration of HF medication is a topic that has received little attention despite its potential to contribute to GDMT utilization and optimization.

### Previous Research on Remote Titration

Several previous trials have investigated remote titration of HF medication with or without the aid of telemonitoring. Two trials by Steckler et al [16] and Moyer-Knox et al [17] assessed remote medication titration over the phone. Steckler et al [16] found that target doses were achieved in 50% of patients for ACEIs or ARBs and in 41% of patients for BBLs. Moyer-Knox et al [17] found that a total of 71% of patients reached target doses of BBLs within approximately 8 weeks. Two other trials have attempted to use telemonitoring for the purpose of remote titration of HF medication. A study by D’Onofrio et al and Palmisano et al [18,19] found that remote BBL titration allowed 76% of patients in the intervention group to achieve target doses versus only 38% of patients in the control group. Similarly, Spaeder et al [20] also performed a study that focused on rapid titration of BBLs and found no statistical difference in the proportion of patients who reached target doses. However, the time frame required to reach target doses was significantly shorter in the intervention group (33.6 vs 63.7 days; *P*<.001). Lastly, a study by Smeets et al [21] attempted to further automate the titration process by incorporating a clinical decision support component. Patients reported high levels of satisfaction and increased medication adherence. However, many technical issues were encountered, no significant differences were observed in the proportion of patients on target doses of BBLs (50% vs 40%; *P*=.69) or ACEIs (42% vs 40%; *P*>.99), and there was no difference in the time taken to uptitrate to guideline-recommended doses. These trials provided preliminary evidence demonstrating that remote titration can be successful and result in a higher proportion of patients reaching target doses within shorter time frames.

### Study Objective

While information and research on remote titration of HF medication is somewhat limited, the results of previous studies have been predominantly positive [16-21]. Building on previous studies of remote titration of BBLs or ACEIs, this study undertakes to investigate remote titration of GDMT triple therapy. An RCT is being conducted with the objective to explore how the combination of remote titration and telemonitoring impacts GDMT optimization compared with standard care. The primary objective of the RCT is to assess the effectiveness of remote titration facilitated by telemonitoring and its impact on the proportion of patients achieving target doses, the time to dose optimization, and the number of visits required to achieve target doses. The secondary objective is to assess the safety of remote titration. This paper reports the findings from an internal pilot [22] of the RCT. As an internal pilot, this study also aims to identify the most suitable primary outcome measure and obtain more accurate data for a sample size calculation.

## Methods

### Study Design Overview

The internal pilot was part of a 2-arm parallel RCT conducted within a mixed-methods study. A detailed description of the full study protocol was published separately [23]. Patients were randomized into an intervention group that relayed physiological and symptom data via a smartphone-based telemonitoring platform (remote titration) or a control group that attended regular clinic visits.

This study received approval from the research ethics boards (REB) of the University of Toronto (REB No. 00036655) and the University Health Network (REB No. 18-5351), where patients were recruited and patient data were stored. The study was also registered at ClinicalTrials.gov (NCT04205513).

### Participants

Study participants were recruited from the Peter Munk Cardiac Centre (PMCC) Heart Function Clinic (HFC). Patients eligible for enrollment were outpatients not yet at target doses of GDMT (ie, ACEIs, ARBs, BBLs, ARNIs, MRAs, or any combination thereof at suboptimal doses), as determined by their cardiologist. Additional inclusion and exclusion criteria are outlined in Textbox 1 and Textbox 2, respectively. Eligible participants were first identified by their cardiologist during their usual HFC visit and invited to speak to a nurse coordinator regarding participation. They met with the nurse coordinator immediately after their HFC visit and underwent the informed consent process. Patients that agreed to participate in the study were requested to sign a consent form.

**Patient inclusion criteria.**

- Able to provide informed consent to participate in the program
- 18 years or older
- Diagnosed with heart failure and followed by a cardiologist at the Peter Munk Cardiac Centre Heart Function Clinic, who has primary responsibility for management of the patient’s heart failure
- New York Heart Association class I-III
- Stable heart failure, defined as no hospitalization within 1 month
- Patient was not yet at target doses of guideline-directed medical therapy (angiotensin-converting enzyme inhibitors, angiotensin receptor blockers, β-blockers, angiotensin receptor–neprilysin inhibitors, mineralocorticoid receptor antagonists, or any combination thereof at suboptimal doses) and hence qualified for uptitration
- Patient or their informal caregiver spoke and read English adequately to participate in the program and understand the Medly app alerts and prompts
- Ability to comply with using Medly (eg, able to stand on the weight scale, able to answer symptom questions, etc)

**Patient exclusion criteria.**

- Active acutely decompensated heart failure
- Already on target doses of guideline-directed medical therapy
- Inability to titrate medications due to adverse events, including:
  - History of angioedema
  - Uncontrolled hypertension
  - Hypotension preventing uptitration
  - Heart rate at rest of <56 beats per minute
- Congenital heart disease
- Previous heart transplant or currently awaiting heart transplant
- Acute coronary syndrome; stroke; transient ischemic attack; cardiac, carotid, or other major cardiovascular surgery; percutaneous coronary intervention; or carotid angioplasty within 6 weeks prior to randomization
- Obstructive or restrictive cardiomyopathy
- Second- or third-degree atrioventricular block without a pacemaker
- Presence of hemodynamically significant mitral or aortic valve disease, except mitral regurgitation
- Presence of other hemodynamically significant obstructive lesions of the left ventricular outflow tract, including aortic and subaortic stenosis, that are not controlled with suitable treatment
- Evidence of hepatic impairment, defined as alanine aminotransferase or aspartate transaminase value more than 3 times the upper normal limit
- Estimated glomerular filtration rate of <30 mL/min/1.73 m^2^ at randomization or >35% decline in estimated glomerular filtration rate between visits
- Known stenosis of both renal arteries
- Hyper- or hypothyroidism not controlled by treatment
- Hyperkalemia of >5.5 mmol/L at randomization
- Hyponatremia of <130 mmol/L at randomization
- History of severe asthma or pulmonary disease
- Presence of any other disease that in the clinician’s opinion would exclude the patient from the study or result in a life expectancy of <1 year

### Medly Telemonitoring Program

Medly, a mobile phone–based telemonitoring program for patients with HF [24,25], was launched at the PMCC HFC in 2016. Medly enables patients to monitor daily weight, blood pressure, heart rate, and symptoms and enter them either manually or via Bluetooth to the Medly app on a mobile phone. The data are then automatically transmitted to a data server. Automated instructions are sent to patients based on a rules-based algorithm that analyzes their measurements and symptoms [26]. Alerts are sent to clinicians and the nurse coordinator in real time if any deterioration is identified. Clinicians can also view alerts and the patients’ telemonitoring data through a secure web portal. Since the Medly program is integrated into the PMCC HFC as part of the standard of care, all patients enrolled into the study were monitored through Medly.

### Interventions

Patients who met the inclusion criteria were enrolled and randomized 1:1 into one of 2 groups: (1) the control group and (2) the intervention group.

The control group underwent standard titration. Participants attended regular titration visits and were provided with the current standard of care, which included the use of Medly. Medication changes were performed based on data collected through assessments performed during clinic visits.

The intervention group underwent remote titration. Participants were called on the phone every 2 weeks to perform medication changes based on Medly data. Patients received requisitions for blood work to be performed at local labs, if required. Patients in the intervention group could still visit the clinic for assessments and follow-ups at their cardiologist’s discretion.

Titration was considered complete when patients reached target GDMT doses specified in the guidelines of the Canadian Cardiovascular Society [4] or maximal tolerated doses, whichever came first.

### Outcome Measures

The primary outcome measure was the number of visits required to achieve target doses. This outcome was assessed based on the number of visits, phone calls, and actions performed at each visit or phone call throughout the study.

Secondary outcome measures included the proportion of patients who achieved target doses, assessed based on the medications and dosages taken by patients at baseline and poststudy as well as all changes made in the medication regimen throughout the study, time to dose optimization, assessed based on the mean and median time to dose optimization in weeks and calculated for each study group, and patient safety outcomes, assessed based on any adverse events (AEs) that occurred throughout the study in each study group. In order to differentiate AEs from symptoms frequently encountered by patients during medication titration, AEs were defined as events that caused titration deferral, dose decreases, changes in the type of medication prescribed, titration termination, or an unscheduled visit to the clinic or visit to the emergency department (ED).

### Sample Size

The sample size for the pilot was calculated based on the outcome measure of the number of visits required to complete titration by using data obtained from the literature [4,11]. Assuming that regular titration visits take place every 2 weeks over 3 to 6 months and anticipating a relative reduction of at least 35% in the number of visits for the intervention group, the sample size for the internal pilot was calculated to be 42. The calculation was performed assuming 80% power, an α of .05 (2-sided), and an attrition rate of 30%. The sample size of the full RCT was subsequently recalculated based on information obtained from this internal pilot, as described in the “Implications for the Full RCT” section.

### Randomization

Patients were randomized 1:1 into control and intervention groups. An online computer-generated randomization tool was used to perform block randomization in blocks of 4 in order to ensure that the treatment groups were as balanced as possible. The generated sequence was used to create randomization envelopes, and the nurse coordinator was provided with the randomly generated treatment allocations within sealed opaque envelopes.

### Statistical Methods

Descriptive, parametric, and nonparametric statistics were performed. McNemar tests were performed on binary baseline and poststudy data, while chi-square tests were performed to compare binary poststudy data between the groups. Independent 2-tailed Student *t* tests and Mann-Whitney tests were performed to compare poststudy data between the groups for normally and nonnormally distributed data, respectively. A *P* value of <.05 was considered significant for all tests. Analysis was performed using the intention-to-treat approach [27] and the IBM SPSS software platform (version 25; IBM Corp).

## Results

### Recruitment

**Figure 1:**
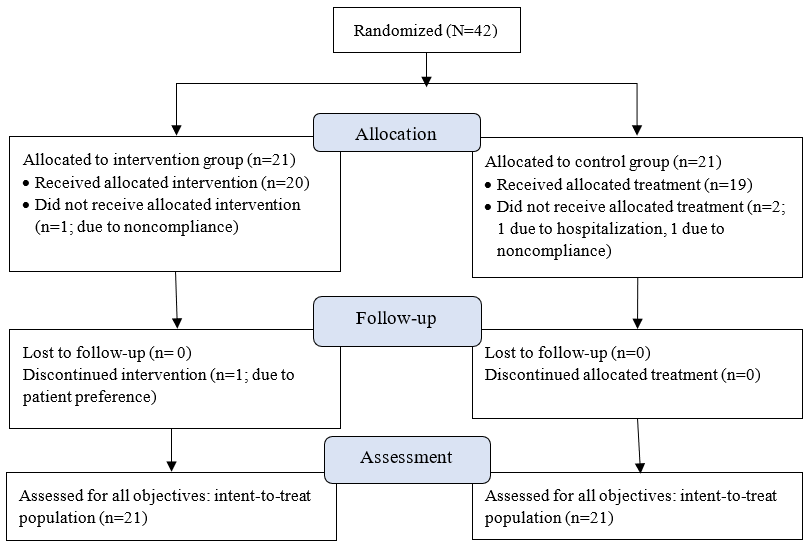
CONSORT diagram of the trial participant flow.

Figure 1 depicts a CONSORT (Consolidated Standards of Reporting Trials) diagram of the trial participant flow. Patients were enrolled into the study between January and June 2019 and followed between January and December 2019. A total of 42 patients were enrolled into the study at baseline. There were 2 patients in each group who did not complete the trial; however, they were included in the data analysis. Reasons for withdrawal included prolonged illness, noncompliance, and patient preference (patient did not wish to be titrated remotely).

### Baseline Data

The baseline demographic and clinical characteristics of the patients are summarized in Table 1. There were more men than women in both groups, which is typical for HFrEF clinics, and the average age of the participants was notably lower than the general HF patient population [28] but was representative of the clinic where the study was conducted. The average age of the patients at the clinic is somewhat younger than the general population of patients with HF because patients are frequently referred to this particular clinic for heart transplant or for mechanical circulatory support device therapy. Therefore, the clinic treats a higher-than-average proportion of severely ill patients compared with other HF clinics, including very young patients with HF. No statistically significant differences were detected between the groups.

**Table 1:** Characteristics of patient participants in the intervention group and control group.

Variable				Intervention group (n=21)	Control group (n=21)
Age (years), mean (SD)				53.00 (10.04)	57.57 (12.21)
**Age range (years), n (%)**					
	30-59			17 (81%)	11 (52%)
	60-79			4 (19%)	10 (48%)
**Gender, n (%)**					
	Male			14 (67%)	16 (76%)
	Female			7 (33%)	5 (24%)
**NYHA^a^** **class, n (%)**					
	I			1 (5%)	3 (14%)
	II			13 (62%)	14 (67%)
	III			7 (33%)	4 (19%)
LVEF^b^, mean (SD)				28.05 (6.65)	27.38 (6.30)
**Primary cause of heart failure, n (%)**					
	Ischemic			9 (43%)	7 (33%)
	Idiopathic			6 (29%)	7 (33%)
	Other			6 (29%)	7 (33%)
**New or existing HF**					
	New-onset HF^c,d^			6 (29%)	4 (19%)
	Existing HF			15 (71%)	17 (81%)
**GDMT^e^** **utilization**					
	**ARNI^f^**				
			ARNI at any dose	3 (14%)	8 (38%)
			ARNI at target dose	1 (5%)	2 (10%)
	**ACEI^g^**				
			ACEI at any dose	13 (62%)	8 (38%)
			ACEI at target dose	4 (19%)	2 (10%)
	**ARB^h^**				
			ARB at any dose	1 (5%)	1 (5%)
			ARB at target dose	0 (0%)	0 (0%)
	**BBL^i^**				
			BBL at any dose	18 (86%)	18 (86%)
			BBL at target dose	5 (24%)	9 (43%)
	**MRA^j^**				
		MRA at any dose		13 (62%)	11 (52%)
		MRA at target dose		4 (19%)	4 (19%)

^a^NYHA: New York Heart Association.

^b^LVEF: left ventricular ejection fraction.

^c^HF: heart failure.

^d^Diagnosed within 3 months of enrollment.

^e^GDMT: guideline-directed medical therapy.

^f^ARNI: angiotensin receptor–neprilysin inhibitor.

^g^ACEI: angiotensin-converting enzyme inhibitor.

^h^ARB: angiotensin receptor blocker.

^i^BBL: β-blocker.

^j^MRA: mineralocorticoid receptor antagonist.

Both patients with new-onset and existing HF were enrolled into the study. As such, it was important to ensure that there were no significant differences in the starting doses of the patients’ GDMT drugs. Table 2 outlines the mean doses of drugs that patients in each of the study groups received at baseline. Drugs prescribed only to 1 patient were not included in the analysis. Mann-Whitney U tests were used to examine differences between the groups whenever possible (n≥5), and no significant differences were identified. Mean starting doses were very similar in both groups for carvedilol and spironolactone, slightly higher in the intervention group for sacubitril-valsartan and ramipril, and slightly higher in the control group for bisoprolol, metoprolol, and perindopril. This variation in starting doses was unlikely to have a substantial impact on study results, particularly since starting doses were more often higher in the control group.

**Table 2:** Mean doses of GDMT drugs prescribed to patients at baseline.

Medication		Intervention group (n=21)				Control group (n=21)				*P* value	
		Participants, n		Dose (mg), mean (SD)		Participants, n		Dose (mg), mean (SD)			
**ARNI^a^**											
	Sacubitril-valsartan		3		116.67 (62.36)		8		100.00 (43.30)		N/A^b^
**β-blocker**											
	Bisoprolol		5		4.50 (3.22)		7		6.25 (3.15)		.19
	Carvedilol		9		16.15 (10.82)		9		15.63 (7.22)		.97
	Metoprolol		4		40.63 (16.24)		2		56.25 (43.75)		N/A
**ACEI^c^**											
	Ramipril		11		4.55 (2.52)		4		2.92 (1.35)		N/A
	Perindopril		2		2.00 (0.00)		4		4.00 (0.00)		N/A
**MRA^d^**											
	Spironolactone		13		19.23 (6.23)		11		21.59 (5.57)		.45

^a^ARNI: angiotensin receptor–neprilysin inhibitor.

^b^N/A: not applicable.

^c^ACEI: angiotensin-converting enzyme inhibitor.

^d^MRA: mineralocorticoid receptor antagonist.

### Number of Visits Required to Achieve Target Doses

From January to December 2019, the intervention group cumulatively had a total of 20 visits and 99 phone calls for the purposes of titration, while the control group had a total of 44 visits. The number of overall patient-clinician contact points was substantially higher in the intervention group. However, when examining clinic visits alone, there was a 54.5% reduction in the intervention group, as the majority of patient-clinician contact points took place over the phone.

On average, the intervention group had 6.3 (SD 2.1) calls and visits and titrated 2.3 (SD 0.65) drugs, while the control group had 2.3 (SD 1.0) visits and titrated 1.6 (SD 0.9) drugs. These differences were statistically significant, with a *P* value of <.001 for the number of calls and visits and a *P* value of .02 for the number of titrated drugs.

### Time to Dose Optimization

**Figure 2:**
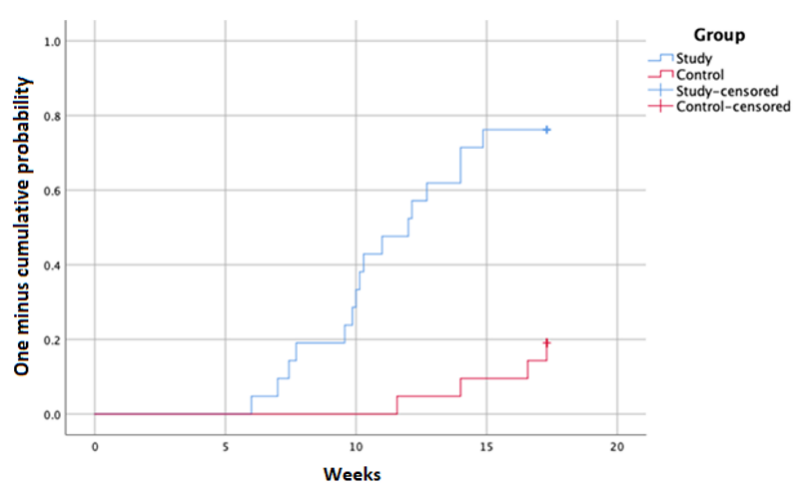
Kaplan-Meier curve of titration completion over a period of 4 months.

The intervention group completed titration within a period of 12.3 (SD 5.0) weeks, with a median of 11.0 weeks, while the control group required 19.0 (SD 4.2) weeks, with a median of 18.8 weeks. A time-to-event analysis was performed to compare titration completion rates between the intervention and control groups over a period of 4 months. Log rank analysis resulted in a *P* value of <.001. The one minus cumulative survival curve was selected to represent the patients that completed titration (Figure 2).

### Proportion of Patients Who Achieved Target Doses

Table 3 outlines the number and proportion of patients who completed titration in each of the groups within various time frames. Analysis was performed once all participants had been in the study for a minimum of six months. Overall, 19 of the 21 patients (90%) in the intervention group and 11 of the 21 patients (52%) in the control group completed titration at the time of analysis (*P*=.002). In addition, 12 of the 21 patients (57%) in the intervention group and 6 of the 21 patients (29%) in the control group achieved triple therapy at target doses (*P*=.003).

**Table 3:** Number and proportion of patients who completed titration within various time frames.

Time frame	Intervention group, n (%) (n=21)	Control group, n (%) (n=21)	*P* value
Within 4 months	16 (76%)	4 (19%)	<.001
Within 5 months	18 (86%)	8 (38%)	.001
Within 6 months	18 (86%)	10 (48%)	.004
Over 6 months	19 (90%)	11 (52%)	.002
Total	19 (90%)	11 (52%)	.002

### Patient Safety Outcomes

The study was not powered to assess AEs. As such, only descriptive statistics were performed on AE data. AEs occurred in 13 of the 21 patients (62%) in the intervention group and 10 of the 21 patients (48%) in the control group. The most common AEs were hypotension, defined as systolic blood pressure below 90 mmHg (11/38, 29% of all events), and dizziness (10/38, 26%), followed by hyperkalemia, defined as potassium levels above 5.5 mmol/L (6/38, 16%), and fatigue (6/38, 16%). No serious AEs or HF-related hospitalizations occurred during the study. One patient in the control group was removed from the per-protocol population due to a lengthy hospitalization that precluded her from undergoing titration. A total of 4 ED visits took place, 2 in each group. One ED visit in the intervention group was a result of suspected atrial fibrillation, while the other ED visits were not associated with cardiovascular issues. Overall, there were no significant differences between the groups and no indications that the AEs were related to the method of titration (ie, remotely or in clinic).

## Discussion

### Summary of Findings

In this pilot RCT of telemonitored remote titration versus usual care, remote titration was associated with a larger proportion of patients achieving target doses, shorter time to optimization, and fewer visits required to achieve target doses.

### Proportion of Patients That Achieved Target Doses

Remote titration increased the proportion of patients achieving target doses within guideline-recommended timeframes (18/21, 86% in the intervention group vs 10/21, 48% in the control group after 6 months of follow-up). The proportion of patients that achieved target doses in our intervention group was notably higher than the numbers outlined in the literature. The CHAMP-HF registry specified that in the general HF population eligible for GDMT, target doses were prescribed to only 17% of patients for ACEIs or ARBs, 14% for ARNIs, 28% for BBLs, and 77% for MRAs [13]. In comparison, our study found that the intervention group demonstrated achievement of target doses at a rate of 38% (8/21) of patients for ACEIs or ARBs, 38% (8/21) for ARNIs, 86% (18/21) for BBLs, and 67% (14/21) for MRAs. In addition, the CHAMP-HF registry noted that only 1% of patients eligible for all classes of medication were receiving target doses of triple therapy, while in our intervention group, 57% (12/21) of patients achieved optimal triple therapy. Only 29% (6/21) of patients in the control group achieved target doses of triple therapy. The titration rates in the control group were still higher than the numbers outlined in the literature. This can be attributed to the fact that the study was performed in a premier heart function clinic with highly experienced cardiologists, where provider-related barriers, such as knowledge of and comfort with the drug therapy optimization, were mitigated. Remote titration also made it possible to mitigate institutional and patient-related barriers, such as time constraints, transportation limitations, and availability of resources necessary to accommodate visits. This may have enabled the intervention group to complete titration in a much timelier fashion.

### Time to Dose Optimization

Our study found that the median time to dose optimization was 11.0 weeks in the intervention group versus 18.8 weeks in the control group, pointing to a nearly 8-week decrease. Similar results were seen in other studies of remote titration. For the time to dose optimization, Steckler et al [16] reported a median time of 54 days, Moyer-Knox et al [17] had a mean time of 42 days, Spaeder et al [20] observed optimal doses with weekly titration over a mean of 33.6 days, and D’Onofrio et al [18] found a mean of 57 days. Overall, our findings fall in line with these studies that primarily examined BBL titration, which comprises about a third of the full GDMT titration process.

Ansari et al [29] noted that in-office, nurse-facilitated medication titration of BBLs achieved 43% titration completion at 12 months. Hickey et al [30] showed that a structured medication titration plan demonstrated a 49% achievement of target doses for ACEIs and ARBs and 46% for BBLs at 6 months. While both of these results were improvements over standard care, they fall short of the results observed in our study, as well as other studies of remote titration.

Time-to-event analysis, presented in Figure 2, outlines the substantial difference in the time to dose optimization between the intervention and control groups. Most patients in the intervention group (16/21, 76%) completed titration within the first 4 months, while only 19% (4/21) of the patients in the control group completed titration within a similar time frame. This is another indicator of the added value that remote titration can introduce into clinical practice. While expert recommendations suggest that clinicians should aim to achieve target doses within 3 to 6 months [11], this timeline is usually quite unfeasible with standard clinic visits [13,14,31,32], and titration may actually take up to 12 months [33]. In contrast, remote titration facilitated by telemonitoring enabled 76% (16/21) of the patients in our intervention group to reach target doses within 4 months and 86% (18/21) within 6 months.

### Number of Visits Required to Achieve Target Doses

Experts recommend titrating medication at 1- to 4-week intervals, depending on the individual patient. As a guideline, the dose can usually be doubled every 2 weeks [4]. However, in practice, such frequent visits may prove unfeasible. Patient constraints and institutional limitations often necessitate spacing visits further apart. In our study, patients in the control group had few visits, while patients in the intervention group had regular phone calls for titration every 2 weeks. Analysis revealed a 2.7-fold increase in the overall number of calls plus clinic visits in the intervention group compared with clinic visits in the control group. Remote titration decreased the number of clinic visits required to achieve target doses by 54.5%. 

This is a very positive finding, since the difficulty in establishing regular and frequent encounters between clinicians and patients has been noted as a significant barrier to GDMT optimization in many studies [31-34]. Patients have substantial time constraints, transportation limitations, or financial limitations that preclude them from being able to attend frequent appointments. Reducing the number of clinic visits while increasing the number of overall patient-clinician contact points allows for timely optimization of GDMT and substantially reduces the financial burden on patients. Furthermore, from an institutional perspective, the availability of infrastructure and resources necessary to accommodate visits is limited. Remote titration could enable remote optimization of more stable patients and free up clinic space and time for patients that require in-person follow-up, thereby contributing to optimal use of clinic resources. Lastly, the reduction in visits could also contribute to distancing, protecting high-risk patients from potential exposure to pathogens that could deteriorate their condition and predispose them to worse outcomes.

### Synthesis of Findings

Guidelines suggest that clinicians should aim to achieve target doses within 3 to 6 months [11]. However, this rapid timeline usually proves unfeasible with standard in-person clinic visits [13,14,31,32]. In practice, optimization of each therapy (ARNIs, ACEIs, or ARBs; BBLs; and MRAs) may require a titration period of 2 to 4 months. With aggressive titration, optimal dosing may be achieved in 6 months; however, in clinical practice it is more likely to take 9 months or potentially up to 12 months [33]. In contrast, remote titration facilitated by telemonitoring enabled 76% (16/21) of patients in our intervention group to reach target doses within 4 months. This points to a significant advantage, especially considering that patients had to attend a minimal number of clinic visits to accomplish this.

The increased proportion of patients who achieved target doses and the shorter timelines observed in this pilot study point to another potential benefit that this intervention could provide to patients with HF. A meta-analysis conducted in 2017 by Zaman et al [35] assessed data from 32,840 patients and calculated the absolute risk of death associated with deferral of HF medical therapy for 1 year. The analysis showed that a 1-year deferral of treatment could reduce the 1-year survival rate from 90% (if treated) to 78% [35]. Our results suggest that remote titration could prevent detrimental therapy optimization delays that can lead to significant disease progression for patients with HF.

### Implications for the Full RCT

As an internal pilot, this study also aimed to inform the choice of the most appropriate primary outcome measure for the full RCT and provide data to contribute to a more accurate sample size calculation. The pilot demonstrated that the initially selected primary outcome measure, the number of clinic visits required to achieve target doses, was strongly influenced by external factors unrelated to the intervention. Furthermore, it did not properly reflect the impact of the intervention on GDMT optimization. The proportion of patients achieving target doses proved to be a central finding that was less susceptible to external factors and served as a good indicator of the utility of remote titration while clearly outlining the differences between the intervention and control groups. Therefore, the new sample size was calculated based on the proportion of patients achieving target doses and determined to be 108 patients [23]. This highlights the importance of internal pilot studies in situations in which there is uncertainty concerning values of such necessary parameters as variances or event rates in the control group [22].

### Study Limitations

The results of this study should be interpreted while taking some limitations into account. The sample size, single-center nature of the study, and availability of dedicated staff to support the intervention may impact its external validity.

The small sample size of the study did not allow any adjustment for possible confounders. Therefore, while the study results are promising, the pilot was not designed with the required power to achieve definitive conclusions regarding effectiveness. While a sample size was calculated based on available data, this pilot aimed to obtain data for a more accurate sample size calculation. *P* values should thus be interpreted accordingly.

The patient population enrolled into this study was recruited from a single specialized heart function clinic that had launched the Medly Program in 2016. First, the familiarity of the clinicians involved in this study with telemonitoring, as well as the existing processes for communication of information obtained through Medly, may have mitigated challenges that could have otherwise been encountered. Second, the intervention was supported by a dedicated nurse coordinator, which might not be available at other clinics, limiting the potential generalizability and external validity of the study. Third, as our study investigates process-of-care changes, blinding could not be applied to physicians. The physicians’ awareness of the group to which their patients were randomized may have impacted their effort to reach target doses. However, as this applied equally to both study groups, the lack of physician blinding is not expected to have a substantial impact on the outcomes of the study. Lastly, the average age of the participants was notably lower than the general HF patient population. As older populations are generally more technophobic, this could reduce the potential generalizability and external validity of the study. However, a previous study conducted with Medly found that its ease of use and the availability of supporting services led to higher use of the app in older patients. Moreover, patients in older age groups (70 years or older) maintained higher and more consistent adherence rates over time [36].

Analysis was performed once all participants had been in the study for a minimum of six months. At the time of analysis, 7 patients in the control group had not yet completed titration. Therefore, the time to dose optimization and number of visits required to achieve titration in the control group represent estimates that are most likely lower than the final numbers. As such, the differences in these parameters represent a conservative assessment, and the actual impact of remote titration on these factors may be larger than presented here.

### Conclusions

A substantial treatment gap exists between guideline-recommended heart failure therapy and the implementation of these guidelines in the clinical care of patients. The results of this pilot study suggest that remote titration facilitated by telemonitoring could be leveraged to garner substantial improvements in GDMT optimization over the standard of care. Remote titration increased the proportion of patients that achieved target doses, decreased the median time to dose optimization, and decreased the number of visits required to achieve target doses. In addition, remote titration may contribute to optimal use of clinic resources by enabling remote therapy optimization for more stable patients while freeing up clinic space and time for patients that require in-person follow-up. Lastly, by facilitating timely optimization of vital therapy for patients with HF and eliminating delays in therapy, remote titration could help reduce preventable disease progression.

## Appendix

Multimedia Appendix 1 CONSORT-EHEALTH checklist (v 1.6.1).

## Abbreviations

ACEI: angiotensin-converting enzyme inhibitors
AE: adverse event
ARB: angiotensin receptor blockers
ARNI: angiotensin receptor–neprilysin inhibitors
BBL: β-blockers
CHAMP-HF: Change the Management of Patients with Heart Failure
CONSORT: Consolidated Standards of Reporting Trials
ED: emergency department
GDMT: guideline-directed medical therapy
HF: heart failure
HFC: Heart Function Clinic
HFrEF: heart failure with reduced ejection fraction
LVEF: left ventricular ejection fraction
MRA: mineralocorticoid receptor antagonists
PMCC: Peter Munk Cardiac Centre
RCT: randomized controlled trial
